# Supplementary material for: Effect of the Gintonin-Enriched Fraction on Glucagon-Like-Protein-1 Release
Source: Molecules. 2021 Oct 18;26(20):6298. doi: 10.3390/molecules26206298 (PMC8539011; doi:10.3390/molecules26206298)
Supplement: Supplementary file 1 [file molecules-26-06298-s001.zip › molecules-1345780-supplementary.pdf]

Supplementary Data

Materials and Methods

Cell Viability Assay

A WST-1 cell proliferation assay (Catalog No. ab155902, Abcam) was used to determine cell viability, as described in an earlier experiment. Briefly, NCI-H716 cells were plated in 96-well microplates at a density of 1 × 10^4 cells/well overnight and treated with GEF (0, 0.1, 0.3, 1, 3, 10, 30, and 100 μg/mL). After 24 h of incubation with GEF, the culture plate was incubated with the WST-1 reagent for 2 h, according to the manufacturer's instructions. Finally, the microplate was scanned for formazan dye detection to measure optical density with a scanning multi-well spectrophotometer (Spectra Max190, Molecular Devices, Sunnyvale) at 570 nm.

Intracellular Ca^{2+} Measurement

Intracellular Ca^{2+} levels in NCI-H716 cells were measured following treatment with different GEF concentrations. Briefly, NCI-H716 cells from each group were incubated for 40 to 60 min at room temperature with 5 μM Fura-2/AM (Molecular Probes, Eugene, OR, USA) and 0.001% Pluronic F-127 (Molecular Probes) in a 4-(2-hydroxyethyl)-1-piperazineethanesulfonic acid (HEPES)-buffered solution (150 mM NaCl, 5 mM KCl, 1 mM $MgCl_2$, 2 mM $CaCl_2$, 10 mM HEPES, and 10 mM glucose) with adjusted to pH 7.4. A xenon arc lamp was used to illuminate cells. For the excitation wavelengths (340 and 380 nm) selection, a computer-controlled filter wheel (Sutter Instruments, Novato, CA, USA) was used and the emitted fluorescence was reflected through a 515 nm filter to a frame transfer-cooled CCD camera (Olympus, Japan). Then a digital fluorescence analyzer was used to calculate the ratios of the emitted fluorescence, being converted to intracellular free Ca^{2+} concentrations $[Ca^{2+}]_i$. All data were analyzed using Universal Imaging software (Bedford Hills, New York, USA).

Western blot using both NCI-H716 Cells and Mouse Intestinal Tissue

NCI-H716 cells and mouse small intestine tissues were treated as described in Materials and Methods 4.3.

Results

GEF-Mediated Cell Viability Assay in NCI-H716 Cells

The cytotoxicity of GEF was determined by WST-1 cell proliferation assay in NCI-H716 cells. GEF (0.1 to 100 μg/mL) did not affect the viability of NCI-H716 cells. GEF 0 μg/mL was set as 100% of control, and relative cell viability decreased as GEF concentration increased. However, no significant difference was observed between the GEF 0 control and GEF treatment group with different concentrations from 0.1 to 100 μg/mL.

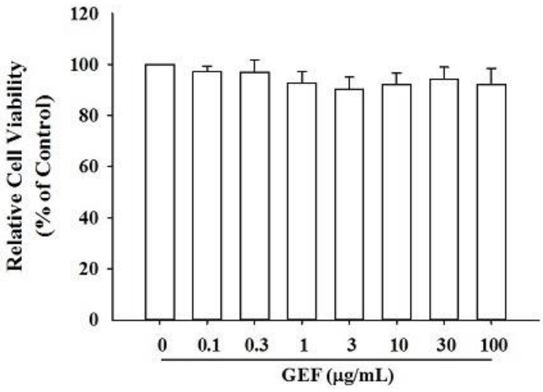

Figure S1. Effects of GEF treatment on cell viability in NCI-H716 cells. **(a)** A cell viability assay was performed following the GEF treatment of NCI-H716 cells. NCI-H716 cells were treated with GEF (0.1, 0.3, 1, 3, 10, 30, or 100 μg/mL) for 24 h. The viability of NCI-H716 cells treated with GEF compared with that of NCI-H716 cells treated with PBS (control). NCI-H716 cell viability was estimated using the WST-1 assay. GEF, gintonin-enriched fraction.

GEF-induced [Ca^{2+}]$_i$ Transient in NCI-H716 Cells

Based on previous studies assessing GEF-induced [Ca^{2+}]$_i$ transients, we hypothesized that NCI-H716 cells would transiently release intracellular calcium following GEF treatment. As shown in Figure S1, GEF treatment did not transiently increase the [Ca^{2+}]$_i$ levels. Low dose GEF treatment (1, 3, 10, or 30 μg/mL) had no effect on intracellular [Ca^{2+}]$_i$ induction, neither did high-dose GEF treatment (100 and 300 μg/mL). In addition, LPA itself also did not induce [Ca^{2+}]$_i$ transients at concentration tested .

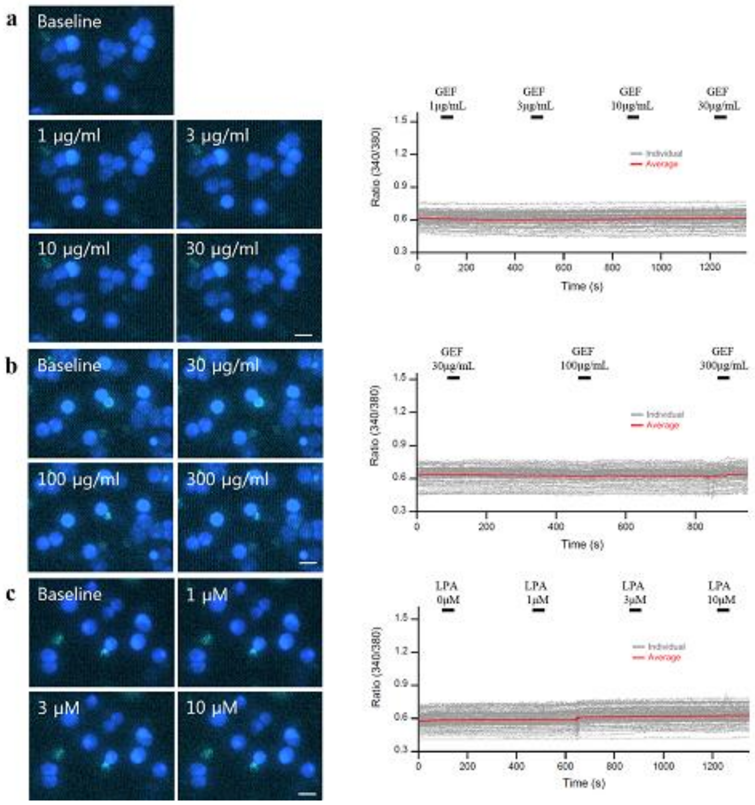

Figure S2. Transient [Ca^{2+}]$_i$ induction in the absence or presence of gintonin-enriched fraction (GEF) at different doses. (a) Lower doses of GEF induce [Ca^{2+}]$_i$ transients in NCI-H716 cells. NCI-H716 cells were treated with low doses of GEF (1, 3, 10, or 30 μg/mL). GEF-mediated [Ca^{2+}]$_i$ transients are not significantly induced. (b) High doses of GEF-induced [Ca^{2+}]$_i$ transients in NCI-H716 cells. NCI-H716 cells were treated with high doses of GEF (30, 100, or 300 μg/mL). GEF-mediated [Ca^{2+}]$_i$ transients are not significantly induced. (c) LPA-mediated calcium influx in NCI-H716 cells. NCI-H716 cells were treated with LPA (1, 3, or 10 μM). LPA-mediated [Ca^{2+}]$_i$ transients are not significantly induced. Data were obtained from 50–80 different cells in three independent experiments. Scale bar = 10 μm

Western blot using both NCI-H716 Cells and Mouse Intestinal Tissue

The same antibodies against 6 LPA receptor subtypes were used both NCI-H716 cells and mouse small intestine tissues. The predicted band sizes were as follows: LPA1 (41kDa-48kDa), LPA2 (39kDa-50kDa), LPA3, (40kDa), LPA4 (42kDa), LPA5 (41kDa), and LPA6 (39kDa).

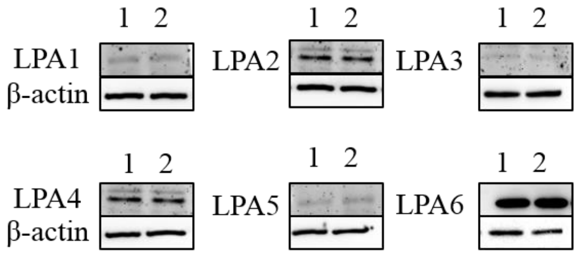

Figure S3. Immunoblotting of LPA receptor subtypes in NCI-H716 cells and mouse small intestine tissues. Western blotting was used to screen the LPA receptor subtypes. 1, NCI-H716 cell extract; 2, mouse small intestine tissue.
